# Psychometric properties of Persian version of wound-QOL questionnaire among older adults suffering from chronic wounds

**DOI:** 10.3389/fpsyg.2022.1041754

**Published:** 2023-01-26

**Authors:** Hamed Savadkoohi, Salman Barasteh, Abbas Ebadi, Hadis Ashrafizadeh, Mahdi Akbarzadeh Amirdehi, Ali Safdari, Mohsen Mollahadi, Saeid Hossein Oghli

**Affiliations:** ^1^Student Research Committee, Baqiyatallah University of Medical Sciences, Tehran, Iran; ^2^Health Management Research Center, Baqiyatallah University of Medical Sciences, Tehran, Iran; ^3^Nursing Faculty, Baqiyatallah University of Medical Sciences, Tehran, Iran; ^4^Behavioral Sciences Research Center, Faculty of Nursing, Baqiyatallah University of Medical Sciences, Tehran, Iran; ^5^Student Research Committee, Faculty of Nursing, Dezful University of Medical Sciences, Dezful, Iran; ^6^The Head of Wound and Ostomy Department, Baqiyatallah Hospital (IIWCC-EWMA-ICW-WOC-ET Nurse), Tehran, Iran; ^7^Student Research Committee, Hamadan University of Medical Sciences, Hamadan, Iran; ^8^Exercise Physiology Research Center, Life Style Institute, Nursing Faculty, Baqiyatallah University of Medical Sciences, Tehran, Iran; ^9^Nursing Care Research Center (NCRC), School of Nursing and Midwifery, Iran University of Medical Sciences, Tehran, Iran

**Keywords:** chronic wound, quality of life, validation, wound-QOL questionnaire, EQ-5D-3L, Iran, Persian, reliability

## Abstract

**Background:**

Patients with chronic wounds experience various biopsychosocial problems which severely affects their quality of life (QoL). Thus, a Persian instrument to assess the QoL of these patients is required. This study aimed to determine the psychometric properties of the Persian version of the wound-QOL questionnaire.

**Methods:**

This methodological study was performed on Iranian patients during 2021–2022. The translation was carried out *via* forward-backward method. Face validity was addressed with 10 patients and content validity with 12 wound specialists. Construct validity was also assessed by performing exploratory factor analysis (EFA) (*n* = 100) and convergent validation with EQ-5D-3L plus Pain VAS Score and known-groups validity. The reliability was assessed by internal consistency using Cronbach’s alpha coefficient and test–retest.

**Results:**

A total of 100 patients with chronic wounds were included in the study. Two factors with cumulative variance of 65.39% were extracted during EFA. The results revealed a significant and high correlation between the total scores of wound-QOL questionnaire, the Persian version of EQ-5D-3L (*p* = 0.000, *r* = 0.502), and Pain score (0–10; *p* = 0.000, *r* = 0.627). The Cronbach’s alpha was 0.743 and stability of the questionnaire (*α* = 0.872) was confirmed. In confirming the known-groups validity, the results showed that this tool can differentiate the QOL of patients with different wounds.

**Conclusion:**

The Persian version of the wound-QOL questionnaire is a valid and reliable questionnaire which can measure the QoL of patients with chronic wounds. This instrument can be used in clinical evaluation as well as research purposes across the Iranian population.

## 1. Introduction

Chronic wounds are wounds that do not heal due to insufficient blood supply, neuropathy, and impaired cell migration; they do not heal over a period of 3 months through a regular physiological healing process in patients with underlying pathology (Li et al., 2003; Werdin et al., 2009). It is estimated that 1–2% of the population will experience chronic wounds during their lifetime in developed countries (Gottrup, 2004). This figure grows with increasing age, since there is a negative relationship between wound healing process and age (Wicke et al., 2009). Estimating wound prevalence, however, is challenging as there is no known agreement or distinction between acute and chronic wounds or a pre-existing consensus for them (Lazarus et al., 1994). However, one study reported that the prevalence of chronic wounds and chronic foot ulcers with various causes was estimated to be 2.21 and 1.51 per 1,000 population, respectively (Martinengo et al., 2019). It is predicted that the global advanced wound care market size would be valued at 18.7 billion dollars (Reportlinker, 2020) by 2027 and 2.6 billion dollars in Asia and Oceania, Australia, India, and South Korea (Sen, 2021). There are no accurate statistics on the status of chronic wounds in Iran. However, a literature review suggests that the prevalence of pressure ulcers as a group of chronic wounds in Iran varies from 3.6 to 45.7% (Afkar et al., 2014; Rafiei, 2016).

Chronic wounds have a significant socio-economic impact due to their frequency, long-term nature, recurrence rate, and social costs (Posnett et al., 2009). Patients with chronic ulcers often have multiple underlying diseases and need a professional caregiver for years. Thus, chronic wounds impose major quality of life (QoL) limitations for patients and their families (Augustin et al., 2017) as well as cause pain, loss of function and mobility, depression, anxiety, social embarrassment and isolation, financial burden, prolonged hospital stay, along with chronic complications or even death (Kloth, 2009).

Health-related QoL (HRQoL) has become a key component in the management of chronic wounds, as it reflects the patient’s view of the disease and treatment (Vowden et al., 2008). Continuous evaluation of HRQoL and its interventions to improve the individual’s conditions is an important aspect of guidance-based wound care (Augustin et al., 2017). QoL is defined by the World Health Organization as “individuals’ perception of their position in life in the context of the culture and value systems in which they live, and in relation to their goals, expectations, standards and concerns.” The purpose of this definition is to express the scope and multidimensional structure of QoL which covers health, social, and economic aspects (Group W, 1997). Healthcare professionals who treat patients suffering from wounds often face the dual challenges of meeting the patient’s expectations for a speedy and trouble-free recovery, as well as early detection of effective treatment settings. However, we also encounter patients whose wounds are incurable and the main goal is to at least maintain or improve the patient’s QoL (Woo et al., 2018). Thus, specific and reliable evaluation instruments are required to include the QoL multidimensionality (Augustin et al., 2017).

So far, several instruments have been introduced to assess wound-QoL, including the Nottingham Health Profile, the Cardiff Wound Impact Schedule (Price and Harding, 2004), the Würzburg Wound (Engelhardt et al., 2014), and the Freiburg Life Quality Assessment for wounds (Augustin et al., 1997). Although these widely used instruments focus on different areas of life and disease-specific injuries, the large number of questions may reduce the tendency to use them in daily practice. Nevertheless, a multinational study has developed a three-part instrument which provides data related to several types of wounds (Augustin et al., 2017). This questionnaire is called wound-QoL and has become a shorter, more practical, and patient-accepted questionnaire. This questionnaire also seems to provide enhanced validation characteristics compared to previous instruments (Augustin et al., 2014). The psychometric properties of wound-QoL have been assessed in several languages, including English, German, French, Italian, Dutch, Swedish, Polish, and Danish, based on international guidelines for developing patient-reported outcomes (PROs; Patrick et al., 2007; Montero et al., 2021). Accordingly, the aim of the present study was to translate and assess psychometric properties of wound-QoL in Iranian population for later use in applied research and evaluation of the results of new therapies as well as generalization of therapeutic effects.

## 2. Materials and methods

### 2.1. Study design

The present methodological study has translated and assessed the psychometric properties of the Persian version of the wound-QOL questionnaire in 2021–2022.

### 2.2. Study population/sampling

The study population included patients with chronic wounds referring to Baqiyatallah Hospital. Inclusion criteria were age least 18 years old, having a chronic wound, informed consent to participate in the study, being literate in Persian, and not having cognitive and mental disorders, according to the individual. Exclusion criteria included the patient’s unwillingness and non-cooperation in completing the questionnaire or incomplete completion of the questionnaire.

### 2.3. Study instruments

#### 2.3.1. Demographic and clinical information questionnaire

A researcher-made questionnaire was used to collect demographic and clinical information such as age, duration of disease (week), body mass index (BMI), sex, marital status, education level, and wound etiology.

#### 2.3.2. Wound-QOL

This questionnaire was developed by Blome et al. (2014), to design and assess psychometric properties of the HRQoL for chronic wounds. The present questionnaire consists of 17 items and 3 sub-scales including everyday life, body, and psyche. The questionnaire items are scored from 1 to 5. The total score is calculated based on the total score of 17 items. The possible score range is 17 and 85 where a higher score indicates a lower QoL score. Its validity was confirmed through content and construct validity. The questionnaire has desired content validity. The three factors of the questionnaire had a cumulative explanation of variance of 51.6%. The instrument reliability was 0.91% by calculating internal consistency and using Cronbach’s alpha coefficient (Blome et al., 2014).

#### 2.3.3. EQ-5D-3L

The EQ-5D was first introduced in 1990 and is one of the most widely used instruments for measuring HRQoL. The EQ-5D-3L version originally consists of two pages: the EQ-5D descriptive system and the visual analog scale.

EQ-5D visual analogue scale (EQ VAS): The EQ-5D-3L descriptive system consists of the following five dimensions, each describing a different aspect: mobility, self-care, usual activities, pain/discomfort, and anxiety/depression. Each dimension has three levels: no problems, slight problems, and severe problems (labeled 1–3). The VAS records the self-rated health of the respondent on a vertical VAS, where it shows the endpoints labeled “Best Health You Can Imagine” and “Worst Health You Can Imagine.” This information can be used as a quantitative measure of the health outcome judged by individual respondents (The Euro Qol Group, 1990).

#### 2.3.4. Pain VAS score

Pain VAS is a continuous scale consisting of a horizontal line (HVAS) or vertical line (VVAS), and is usually 10 cm (100 mm) long. Pain VAS is a single-item scale on which pain intensity scores are usually recorded from “painless” (Score = 0) and “worst pain imaginable” (Score = 100; Hawker et al., 2011).

### 2.4. Translation procedure

Forward-backward translation was carried out. In the forward translation stage, the original English version of the wound-QOL questionnaire was translated into Persian by two specialized English translators after obtaining permission from its developers, according to the International Quality of Life Assessment (IQOLA) protocol (Bullinger et al., 1998). Then, the two translated versions of the questionnaire were reviewed, and finally in a meeting with the researchers the final version was made. Finally, an initial joint translation was obtained based on the agreement of the researchers. In the backward translation stage, the joint Persian translation, which was prepared in the previous stage, was translated into English by two native Persian and English speakers, where an English version was obtained. For comparison, the two English translations obtained in the previous step were sent to the questionnaire developer. The questionnaire was sent by the developer and was then compared with the original version of the questionnaire and approved conceptually.

### 2.5. Face and content validity

After the translation process, cognitive interviews were performed to assess the qualitative face validity. Face validity is defined as the degree respondents or users judge that the items of an assessment instrument are appropriate to the targeted construct and assessment objectives (Sirati Nir et al., 2022). A cognitive interview is conducted with the aim of identifying the source of errors in the questionnaire by focusing on the cognitive processes of respondents when filling out the questionnaire (Willis, 2004). Accordingly, interviews were conducted with ten patients with chronic wounds as well as different economic status and social education. They were asked to rate the legibility, clarity, structure of the questions, ease of comprehension, item difficulty, confusing words, classification of the questions, ease of answering, language forms, and wording. Then corrections were applied in the Persian version of wound-QOL. To check the content validity, the Persian version of the questionnaire was given to 12 experts in the field of chronic wounds, and they were asked to check grammar, use of appropriate words, proper placement of items, and proper scoring (Cook and Beckman, 2006). Also, to assess the content validity, the floor and ceiling effects were assessed. There is a ceiling or floor effect when more than 15% of the respondents obtain the highest or lowest attainable score, respectively. The ceiling or floor effect of more than 15% indicates that the items showing the maximum or minimum intensity of the phenomenon are probably not included, which suggests insufficient content validity of the instrument (Terwee et al., 2007).

### 2.6. Construct validity

Three methods of exploratory factor analysis (EFA), convergent validity, and known-groups validity were used to evaluate the construct validity of this tool.

#### 2.6.1. Exploratory Factor Analysis (EFA)

EFA is used to discover the underlying structure of a relatively large set of variables. The minimum sample size required for EFA is 3–10 participants per item (Kellar and Kelvin, 2013). To evaluate EFA, 100 patients with chronic wounds were included in the study by convenience sampling method. Keiser-Meyer-Olkin test (KMO) and Bartlett’s test were performed to evaluate the adequacy of sampling and suitability of the samples. A KMO value of closer to one is more suitable for factor analysis, but in general, score >0.5 and >0.7 are acceptable and more appropriate, respectively (Ebadi et al., 2017). A value of *p* < 0.05 is accepted in Bartlett’s test (Kaiser, 1974; Hair, 2009). The appropriate results of KMO and Bartlett’s tests indicate the existence of a favorable correlation matrix for factor analysis (Mohammadbeigi et al., 2015). Factor loading refers to the relationship between each factor and each question of the questionnaire; in order for each question to remain in the questionnaire, that relationship must be appropriate. The minimum factor loading in the present study was considered 0.3 where factor loading less than 0.3 indicates a poor relationship between factor and question (Hair et al., 2010; Rashidi Fakari et al., 2020). Factor extraction and interpretation were carried out using principal component analysis and PROMAX rotation, respectively (Samitsch, 2014).

#### 2.6.2. Convergent validation

The second method to evaluate the construct validity in the present study was convergent validation (Krabbe, 2016). Respondents simultaneously answered EQ-5D-3L and Pain score (0–10) along with the Persian version of the wound-QOL scale. To confirm this method, the correlations between wound-QOL scale and EQ-5D descriptive system as well as visual analog scale, EQ-5D visual analogue scale (EQ VAS), and pain score (0–10) were measured using Pearson correlation coefficient.

#### 2.6.3. Known-groups validity

To assess construct validity in the present study, known-groups validity was investigated. This type of construct validity indicates the ability of the test to discriminate between the two groups or more considering the examined construct. One-way analysis of variance (ANOVA) assessed statistical differences in wound-QOL across the four types of wounds (diabetic, pressure, surgical, and others).

### 2.7. Reliability

In order to determine the questionnaire reliability, internal consistency and stability were assessed. Cronbach’s alpha coefficient was calculated to measure internal consistency. To ensure good internal consistency, Cronbach’s alpha is between 0.7 and 0.8 (Rattray and Jones, 2007). In order to determine the instrument stability, the test-re-test method was used (sample size = 30 people). In this study, the interval between test–retest was 14 days and the scores obtained in these two stages were compared using the intraclass correlation coefficient (ICC) test. ICC index above 0.80 is assumed as the desired stability (De Boer et al., 2004). The total-item correlation was also investigated in the present study. The correlation between each question and the total score of the scale was compared after which decisions were made as to whether remove or keep the question, accordingly. Questions with a correlation (*r*) less than 0.3 were excluded (Stevens, 2012).

### 2.8. Ethical considerations

The study was approved by the Ethics Committee of Baqiyatallah University of Medical Sciences with the ethics code IR.BMSU.REC.1400.093. After obtaining written permission *via* email from the instrument developer, the translation process was performed. Prior to the study, the participants were informed of the objectives of the research and participated in the research with written informed consent. They were also informed that all data in the study would remain confidential and that they would be able to withdraw from the study at any time.

## 3. Results

### 3.1. Study population/sampling

A total of 100 patients with chronic wounds were included in the present study. The age of patients ranged from 18 to 93 years (mean age: 65.24 ± 18.88). A total of 66% of patients were male and 34% were female. The mean BMI was 26.14 ± 5.41 kg/m^2^. The mean ± SD duration of wound healing was 15.5 ± 19.99. Most patients were married (68%). Also, about half of the patients had a diploma degree (49%). The prevalence of diabetic, pressure, surgical, and other wounds was 24, 45, 17 and 14%, respectively (Table 1).

**Table 1:** Demographic and clinical characteristics of the study samples.

Variable	*n*	Mean ± SD
Age (years)	100	65.24 (18.88)
Duration (weeks)	100	15.5 (19.99)
BMI	100	26.14 (5.41)
	*n*	%
*Gender*		
Male	66	66
Female	34	34
*Marital status*		
Single	12	12
Married	68	68
Divorce/widow	20	20
*Education*		
Elementary	19	19
Diploma	49	49
Academic	32	32
*Wound etiology*		
Diabetic ulcers	24	24
Pressure ulcers	45	45
Surgical	17	17
Others	14	14

### 3.2. Face and content validity

When assessing face validity, items did not change due to their simplicity and clarity. The qualitative content validity was confirmed using the opinions of 12 chronic wound care experts. Minor changes were made to the text of the questions. The floor and ceiling effects were equal to 2 and 3%, respectively.

### 3.3. Construct validity

#### 3.3.1. EFA

A KMO value of 0.876 was obtained, and Bartlett’s test of sphericity was significant (X2 = 1732.83, df = 136, *p* = 0.000). Two factors were extracted, and named as “Physical ailments” and “Everyday life.” These two factors explained 65.39% of the total variance of wound-QOL (Table 2).

**Table 2:** Exploratory factor analysis of the Farsi version of the wound-QOL.

Factor	Items	Factor loading %	Variance
Factor1 (body/physical ailments)	5	0.956	47.51
	6	0.951	
	8	0.951	
	7	0.939	
	4	0.906	
	9	0.898	
	3	0.709	
	10	0.638	
	1	0.598	
	2	0.538	
Factor2 (everyday life)	13	0.985	17.88
	12	0.918	
	14	0.896	
	15	0.737	
	11	0.658	
	16	0.563	
	17	0.306	
Cumulative%	65.39		

### 3.4. Convergent validity

To assess the convergent validity, there was a significant correlation between the total scores of the respondents to the Persian version of the wound-QOL questionnaire and the Persian version of EQ-5D-3L (*p* = 0.000, *r* = 0.502), EQ-5D-3L and VAS version (*p* = 0.027 and *r* = 0.222), and pain score (0–10; *p* = 0.000 and *r* = 0.627). In other words, the higher wound-QoL score, the greater the score of the EQ-5D descriptive system, EQ VAS will be. Also, the more pain the patient endures, the higher the wound-QoL score will be (Table 3).

**Table 3:** Convergent validity correlation of the wound-QOL global score with concurrent criteria.

	Total wound-QOL		Body/physical ailments		Everyday life	
	*p*	*r*	*p*	*r*	*p*	*r*
EQ-5D-3L*	0.000	0.502	0.000	0.420	0.000	0.430
EQ-5D-3L (VAS**) (0–100)	0.027	0.222	0.009	0.26	0.473	0.073
Pain score (0–10)	0.000	0.627	0.000	0.77	0.133	0.151

*European quality of life 5 dimensions 3 level version. **Visual analogue scale.

### 3.5. Known-groups validity

The results revealed that this tool can differentiate the QOL of patients with different wounds. The value of p of Levene’s test was <0.05. As a result, the assumption of equality of variances in wound-QoL was confirmed. Also, based on the resulting *F* values, it is clear that the null hypothesis, that is, mean equality between different wounds is rejected. *p*-value (sig) was less than 0.05. As a result, there has been a significant difference between at least two types of wounds in terms of QoL score. The results of the present study indicated a significant difference between the QoL of pressure and surgical wounds (*P* = 0.001), pressure and other wounds (*p* = 0.030), as well as between surgical and diabetic wounds (*p* = 0.055).

### 3.6. Reliability

A test–retest was used to evaluate the stability reliability of the questionnaire. The total stability reliability of the questionnaire (ICC = 0.743), body/physical ailments factor (ICC = 0.641), and everyday life factor (ICC = 0.748) was confirmed. To assess the internal consistency of the questionnaire, Cronbach’s alpha coefficient was investigated. The total internal consistency was confirmed for the entire questionnaire (*α* = 0.872), body/physical ailments factor (*α* = 0.866), and everyday life factor (*α* = 0.778).

## 4. Discussion

The QoL of patients with chronic wounds provides useful information about the conditions of these patients to health service providers. An important cornerstone of evidence-based care of chronic wounds is to use a questionnaire to continuously assess the QoL and its interventions in order to improve the individual’s condition. Thus, the aim of the present study was to determine the psychometric properties of the Persian version of the wound-QoL questionnaire in patients with chronic wounds.

According to the results, the face and content validity of the above instrument were confirmed by experts. Also, known-groups validity, EFA, and convergent validity were also used to assess the construct validity, with the results indicating that the studied instrument has a suitable structure. The instrument reliability was calculated by internal consistency method (*α* = 0.872), which revealed the appropriate reliability of the instrument. Test–retest reliability was also obtained (ICC = 0.743) using Pearson correlation coefficient, which confirmed the stability reliability of the instrument.

The translation process was carried out carefully until a final Persian version was obtained. The face validity of the instrument was assessed based on the opinions of 10 patients with chronic wounds, where the results showed that the items are simple and clear. Similar to the results of the present study, the face and content validation processes were performed in the Spanish version of the scale (sample size = 10 patients with chronic wounds) by Montero et al. (2021). Further, there were minor changes in responses of the item “Wound has affected my sleep” of the Dutch version of the above instrument; so that this item retains the same meaning (Amesz et al., 2020). The face and content validity of the above instrument has also been assessed and confirmed in the United States and Germany (Augustin et al., 2017; Sommer et al., 2020), which showed that the items were simple and clear following the translation process.

Known-groups validity, EFA, and convergent validation were performed using EQ-5D-3L and Pain score scales (0–10) to confirm the construct validity in the present study. With regard to results of EFA, that is the extraction of two factors (body/physical ailments, everyday life), the present study has been similar to the studies by Augustin et al. on 227 patients with ulcer in the German version (Augustin et al., 2017), but it has been different from Sommer et al. studies on 599 patients in the American version (Sommer et al., 2020) and Montero et al. in the Dutch version (Montero et al., 2021) by extracting three factors (body, psyche, and everyday life). This difference is probably due to the variety in the types of chronic wounds and the difference in sample size. Indeed, the sample size of our study has been similar to the study of Augustin et al., while on the other hand the sample size of Sommer et al.’s study was several times that of the present study. Also, in our study, the main etiology of the wound was related to pressure ulcer, while in the study of Montero et al. and Sommer et al., venous ulcer constituted the main etiology.

In order to evaluate the convergent validity, the respondents simultaneously responded to the EQ-5D-3L and Pain score scales (0–10) along with the wound-QoL scale. Pearson’s correlation coefficient showed that coefficient of wound-QoL scale with the EQ-5D descriptive system scale and EQ VAS was 0.502 (*r* = 0.502, *p* = 0.000) and 0.222 (value of *p* < 0.027), respectively. In other words, there has been a significant relationship between these two QoL scores. Also, there was a significant relationship between wound-QoL scale and Pain score scale (0–10; *r* = 0.627, *p* = 0.000). In other words, the more pain the patient endured, the higher wound-QoL score will be, and the individual has a lower QoL score. The results of this section showed an appropriate correlation and confirmed the convergent validity of this scale. In the study of Amesz et al., convergent validity was confirmed using wound-QoL, EQ-5D-3L (a generic questionnaire to measure HRQoL), and a visual analog scale (VAS; Amesz et al., 2020). Further, the results of the present study have been similar to those of the Swedish version (Fagerdahl and Bergström, 2018), in which the convergent validity was confirmed using HRQoL, EQ-5D-3L, and EQ-VAS instruments (Augustin et al., 2017). In the American translation, convergent validity was also confirmed using other instruments including wound-QoL and pain, the surface area of the largest wound, total surface area, and the total number of active wounds (Sommer et al., 2020).

The results of the present study revealed a significant correlation between the wound-QOL instrument and the pain score. In other words, the more pain the patient endures, the higher wound-QoL score will be and the individuals has a lower QoL score. Other studies have described pain as a negative factor affecting HRQoL (González-Consuegra and Verdú, 2011; Hopman et al., 2013). The results of the multivariate regression model in a previous study referred to pain as a predictor of negative changes in the overall HRQoL of patients with chronic wounds, as well as in three of the four subscales of FPQLI-WV (Ferrans and Powers Quality of Life Index-Wound version), which are in line with the relevant literature (Silva and Carmo, 2008; González-Consuegra and Verdú, 2011). The results of the study by Amesz et al. showed a significant yet moderate correlation between wound-QoL and VAS pain, and explained that wound pain is not the only characteristic that affects disease-specific HRQoL (Amesz et al., 2020). Note that pain may be caused not only by the wound itself, but also by wound-related factors, such as wound dressing (Price et al., 2008). Sommer et al. found a significant relationship between criteria such as wound pain and odor (Sommer et al., 2020). Oliveira et al. reported a relationship between pain intensity and three areas: “well-being,” “physical symptoms and daily life,” and “social life” (Oliveira et al., 2019). Other studies have highlighted that pain has negative impacts on QoL, since it causes discomfort and limits the activities of daily as well as social life (Dias et al., 2014; Deufert and Graml, 2017; Santos et al., 2017). However, differences between these constructs indicate that general HRQoL is affected by aspects other than wound.

Known-groups validity was used to differentiate between QoL of two or more groups of patients with chronic wounds. The results of this test indicated that this instrument can differentiate QoL of patients with different wounds. The results of the present study showed a significant difference between the QoL of pressure and surgical wounds, pressure, and other wounds as well as between surgical and diabetic wounds. Oliveira et al. also found a statistically significant difference between QoL of patients with pressure ulcers (PU) and traumatic wounds where wounds had the greatest impact on “well-being” as a one of QoL domains (Oliveira et al., 2019).

The present study also showed that floor and ceiling effect was 2 and 3%, respectively. In the main version of this scale, a low floor effect was observed in T1: 0.5%, T2: 1%, T3: 4%, while low ceiling effects were also (0, 1, 0.5%, respectively; Augustin et al., 2017). In the Spanish version, the floor and ceiling effects were low for T1 and higher floor effects were found at T2, which could be interpreted as a positive effect of treatment on HRQoL impairment and, may therefore, indicate a good wound-QoL response to clinical improvement (Montero et al., 2021). In addition to the wound-QoL scale characteristics in terms of mean and standard deviation, floor and ceiling effects in the English version of wound-QoL United States showed a slight deviation to the left, which favors higher HRQoL within the range 0–4 0 indicates the highest quality (Sommer et al., 2020). In the Dutch version, the global score revealed no ceiling effect at any temporal point and only a slight floor effect at T1: 0.8%. Although the “body” and “psyche” subscales showed no ceiling effect at T0, the “psyche” subscale indicated a slight ceiling effect at T1: 0.8%. The “everyday life” subscale showed slight ceiling effects at both temporal points (T0: 1.7%, T1: 3.4%). All subscales showed floor effects in both T0 (body: 12.5%, psyche: 9.2%, everyday life: 6.7%) and T1 (21.7, 7.5, 11.1, respectively; Amesz et al., 2020).

In the present study, the reliability measurement of the Persian version of the wound-QoL scale was confirmed using internal consistency (*α* = 0.872) and stability (Test–retest = 0.743), which indicated the appropriate reliability of the instrument. This result was similar to the findings of the original version of the wound-QoL scale (*α* = 0.928; Augustin et al., 2017). Also, similar to the results of studies by Montero et al., Sommer et al., and Amesz et al., who aimed to carry out cross cultural adaptation of the Spanish version (*α* ≥ 0.80; Montero et al., 2021), the American version (*α* = 0.92; Sommer et al., 2020), and the Dutch version of the Wound-QoL questionnaire, the results of the present study on the reliability of Wound-QoL global score were high at both time points (T0: Cronbach’s *α* = 0.89, T1: Cronbach’s *α* = 0.92; Amesz et al., 2020).

## 5. Study advantages and limitations

The most important advantage of the present study was the use of several methods to evaluate the construct validity including EFA, convergent validity, and known-group validity plus reliability using test–retest and internal consistency methods. Nevertheless, the most important limitation of the present study, as with most cross-sectional studies, was convenience sampling such that we included all patients with chronic wounds in the study. Other limitations of the present study included access to patients with chronic wounds from one treatment center. Thus, selection bias cannot be eliminated. Moreover, there may be a group of wounds that are difficult to heal.

## 6. Conclusion

The results of the present study revealed that the Persian version of wound-QoL is a valid and applicable tool with high patient acceptance for HRQoL evaluation in clinical care and clinical trials in Iran. Another advantage of the instrument in question is its brevity. This instrument is widely validated and its versions are available in different languages. This instrument can also be used in patients with chronic wounds and various medical centers such as hospitals, nursing homes, and wound clinics. Thus, continuous wound-QoL testing in clinical practice may help improve patient-centered care thus enhancing the HRQoL of patients with non-healing wounds.

## 7. Suggestion for further studies

We suggest that the psychometric properties of this tool be examined in a larger sample size. We also recommend that the study be done in various settings such as home care, nursing homes, as well as long-term care facilities and hospices.

## Data availability statement

The raw data supporting the conclusions of this article will be made available by the authors, without undue reservation.

## Ethics statement

The studies involving human participants were reviewed and approved by Research Committee of Baqiyatallah University of Medical Sciences. The patients/participants provided their written informed consent to participate in this study.

## Author contributions

SB and AE: conception, design, analysis and interpretation and statistical analysis. HS, MA, MM, and AS: data collection. SH, SB, and HA: writing and critical revision of the article. HS, SB, AE, HA, MA, AS, and SH: final approval of the article. Not applicable: obtained funding. SH: overall responsibility. All authors contributed to the article and approved the submitted version.

## Conflict of interest

The authors declare that the research was conducted in the absence of any commercial or financial relationships that could be construed as a potential conflict of interest.

The reviewers SS and MT declared a shared affiliation with the author SO to the handling editor at the time of review.

## Publisher’s note

All claims expressed in this article are solely those of the authors and do not necessarily represent those of their affiliated organizations, or those of the publisher, the editors and the reviewers. Any product that may be evaluated in this article, or claim that may be made by its manufacturer, is not guaranteed or endorsed by the publisher.

## References

[ref1] Afkar A. Mahboubi M. Mehrabian F. Farmanbar R. Ghahramani F. Nezhad E. K.. (2014). Predictive factors of ICU bedsores using Braden scale. J. Kermanshah Univ. Med. Sci. 18, 220–225. doi: 10.22110/jkums.v18i4.1855

[ref2] Amesz S. F. Klein T. M. Meulendijks A. M. Nguyen T.-V. Blome C. Roodbol P. F.. (2020). A translation and preliminary validation of the Dutch wound-QoL questionnaire. BMC Dermatol. 20, 5–9. doi: 10.1186/s12895-020-00101-2, PMID: 32843014 PMC7449034

[ref3] Augustin M. Baade K. Herberger K. Protz K. Goepel L. Wild T.. (2014). Use of the wound QoL instrument in routine practice: feasibility, validity and development of an implementation tool. Wound Med. 5, 4–8. doi: 10.1016/j.wndm.2014.04.001

[ref4] Augustin M. Conde Montero E. Zander N. Baade K. Herberger K. Debus E. S.. (2017). Validity and feasibility of the wound-QoL questionnaire on health-related quality of life in chronic wounds. Wound Repair Regen. 25, 852–857. doi: 10.1111/wrr.12583, PMID: 29080332

[ref5] Augustin M. Dieterle W. Zschocke I. Brill C. Trefzer D. Peschen M.. (1997). Development and validation of a disease-specific questionnaire on the quality of life of patients with chronic venous insufficiency. VASA Z. Gefasskrankheiten. 26, 291–301. PMID: 9409180

[ref6] Blome C. Baade K. Sebastian Debus E. Price P. Augustin M. (2014). The “wound-QoL”: a short questionnaire measuring quality of life in patients with chronic wounds based on three established disease-specific instruments. Wound Repair Regen. 22, 504–514. doi: 10.1111/wrr.12193, PMID: 24899053

[ref7] Bullinger M. Alonso J. Apolone G. Leplège A. Sullivan M. Wood-Dauphinee S.. (1998). Translating health status questionnaires and evaluating their quality: the IQOLA project approach. J. Clin. Epidemiol. 51, 913–923. doi: 10.1016/S0895-4356(98)00082-1 9817108

[ref8] Cook D. A. Beckman T. J. (2006). Current concepts in validity and reliability for psychometric instruments: theory and application. Am. J. Med. 119:166.e7. doi: 10.1016/j.amjmed.2005.10.036, PMID: 16443422

[ref9] De Boer M. R. Moll A. C. De Vet H. C. Terwee C. B. Völker-Dieben H. J. Van Rens G. H. (2004). Psychometric properties of vision-related quality of life questionnaires: a systematic review. Ophthalmic Physiol. Opt. 24, 257–273. doi: 10.1111/j.1475-1313.2004.00187.x, PMID: 15228503

[ref10] Deufert D. Graml R. (2017). Disease-specific, health-related quality of life (HRQoL) of people with chronic wounds – a descriptive cross-sectional study using the wound-QoL. Wound Med. 16, 29–33. doi: 10.1016/j.wndm.2017.01.006

[ref11] Dias T. Y. A. F. Costa I. K. F. Melo M. D. M. Torres S. M. S. G. S. Maia E. M. C. Torres G. V. (2014). Quality of life assessment of patients with and without venous ulcer. Rev. Lat. Am. Enfermagem 22, 576–581. doi: 10.1590/0104-1169.3304.2454, PMID: 25296140 PMC4292650

[ref12] Ebadi A. Zarshenas L. Rakhshan M. Zareiyan A. Sharifnia S. Mojahedi M. (2017). Principles of Scale Development in Health Science. Tehran: Jame-e-Negar.

[ref13] Engelhardt M. Spech E. Diener H. Faller H. Augustin M. Debus E. S. (2014). Validation of the disease-specific quality of life Wuerzburg wound score in patients with chronic leg ulcer. Vasa 43, 372–379. doi: 10.1024/0301-1526/a000378, PMID: 25147014

[ref14] Fagerdahl A.-M. Bergström G. (2018). Translation and validation of a wound-specific, quality-of-life instrument (the wound-QoL) in a Swedish population. Ostomy Wound Manage 64, 40–46. doi: 10.1111/scs.12050, PMID: 29847310

[ref15] González-Consuegra R. V. Verdú J. (2011). Quality of life in people with venous leg ulcers: an integrative review. J. Adv. Nurs. 67, 926–944. doi: 10.1111/j.1365-2648.2010.05568.x, PMID: 21241355

[ref16] Gottrup F. (2004). A specialized wound-healing center concept: importance of a multidisciplinary department structure and surgical treatment facilities in the treatment of chronic wounds. Am. J. Surg. 187, S38–S43. doi: 10.1016/S0002-9610(03)00303-9 15147991

[ref17] Group W. (1997). Measuring Quality of Life. Geneva: The World Health Organization. 347–350.

[ref18] Hair J. F. (2009). Multivariate Data Analysis: A Global Perspective. 7th Edn. Upper Saddle River: Prentice Hall.

[ref19] Hair J. F. Black W. C. Babin B. J. (2010). Multivariate data analysis: A global perspective: Pearson Education, Upper Saddle River (N.J.): Pearson education.

[ref21] Hawker G. A. Mian S. Kendzerska T. French M. (2011). Measures of adult pain: visual analog scale for pain (vas pain), numeric rating scale for pain (nrs pain), mcgill pain questionnaire (mpq), short-form mcgill pain questionnaire (sf-mpq), chronic pain grade scale (cpgs), short form-36 bodily pain scale (sf-36 bps), and measure of intermittent and constant osteoarthritis pain (icoap). Arthritis Care Res. 63, S240–S252. doi: 10.1002/acr.20543, PMID: 22588748

[ref22] Hopman W. Buchanan M. Van Den Kerkhof E. Harrison M. (2013). Pain and health-related quality of life in people with chronic leg ulcers. Chronic Dis. Inj. Can. 33, 167–174. doi: 10.24095/hpcdp.33.3.07 23735456

[ref23] Kaiser H. F. (1974). An index of factorial simplicity. Psychometrika 39, 31–36. doi: 10.1007/BF02291575

[ref24] Kellar S. P. Kelvin E. A. (2013). Munro’s Statistical Methods for Health Care Research Wolters Kluwer Health/Lippincott Philadelphia: Williams & Wilkins.

[ref25] Kloth L. (2009). The roles of physical therapists in wound management, part II: patient and wound evaluation. J. Am. Col. Certif. Wound Spec. 1, 49–50. doi: 10.1016/j.jcws.2009.03.003, PMID: 24527113 PMC3478920

[ref26] Krabbe P. (2016). The Measurement of Health and Health Status: Concepts, Methods and Applications from a Multidisciplinary Perspective. San Diego: Academic Press.

[ref27] Lazarus G. S. Cooper D. M. Knighton D. R. Margolis D. J. Percoraro R. E. Rodeheaver G.. (1994). Definitions and guidelines for assessment of wounds and evaluation of healing. Wound Repair Regen. 2, 165–170. doi: 10.1046/j.1524-475X.1994.20305.x, PMID: 17156107

[ref28] Li G. Gustafson-Brown C. Hanks S. K. Nason K. Arbeit J. M. Pogliano K.. (2003). C-Jun is essential for organization of the epidermal leading edge. Dev. Cell 4, 865–877. doi: 10.1016/S1534-5807(03)00159-X, PMID: 12791271

[ref29] Martinengo L. Olsson M. Bajpai R. Soljak M. Upton Z. Schmidtchen A.. (2019). Prevalence of chronic wounds in the general population: systematic review and meta-analysis of observational studies. Ann. Epidemiol. 29, 8–15. doi: 10.1016/j.annepidem.2018.10.005, PMID: 30497932

[ref30] Mohammadbeigi A. Mohammadsalehi N. Aligol M. (2015). Validity and reliability of the instruments and types of measurments in health applied researches. J. Rafsanjan Univ. Med. Sci. 13, 1153–1170.

[ref31] Montero E. C. Sommer R. Augustin M. Blome C. Martínez R. C. Reales C. H.. (2021). Validation of the Spanish wound-QoL questionnaire. Actas Dermosifiliogr. 112, 44–51. doi: 10.1016/j.adengl.2020.11.004, PMID: 33137321

[ref32] Oliveira A. C. Rocha D. M. Bezerra S. M. G. Andrade E. M. L. R. Santos A. M. R. Nogueira L. T. (2019). Quality of life of people with chronic wounds. Acta Paulista Enfermagem. 32, 194–201. doi: 10.1590/1982-0194201900027

[ref33] Patrick D. L. Burke L. B. Powers J. H. Scott J. A. Rock E. P. Dawisha S.. (2007). Patient-reported outcomes to support medical product labeling claims: FDA perspective. Value Health 10, S125–S137. doi: 10.1111/j.1524-4733.2007.00275.x, PMID: 17995471

[ref34] Posnett J. Gottrup F. Lundgren H. Saal G. (2009). The resource impact of wounds on health-care providers in Europe. J. Wound Care 18, 154–161. doi: 10.12968/jowc.2009.18.4.41607, PMID: 19349935

[ref35] Price P. E. Fagervik-Morton H. Mudge E. J. Beele H. Ruiz J. C. Nystrøm T. H.. (2008). Dressing-related pain in patients with chronic wounds: an international patient perspective. Int. Wound J. 5, 159–171. doi: 10.1111/j.1742-481X.2008.00471.x, PMID: 18494622 PMC7951668

[ref36] Price P. Harding K. (2004). Cardiff wound impact schedule: the development of a condition-specific questionnaire to assess health-related quality of life in patients with chronic wounds of the lower limb. Int. Wound J. 1, 10–17. doi: 10.1111/j.1742-481x.2004.00007.x, PMID: 16722893 PMC7951606

[ref37] Rafiei H. (2016). Incidence of pressure ulcer in patients who were admitted to open heart cardiac surgery intensive care unit. Int. J. Epidemiol. Res. 3, 12–18.

[ref38] Rashidi Fakari F. Ebadi A. Ozgoli G. Kariman N. Mohamadizeidi B. (2020). Evaluation of psychometric properties of Persian version of geriatric mistreatment scale in the elderly living in Tehran in 2017-2018: a descriptive study. J. Rafsanjan Univ. Med. Sci. 19, 265–278. doi: 10.29252/jrums.19.3.265

[ref39] Rattray J. Jones M. C. (2007). Essential elements of questionnaire design and development. J. Clin. Nurs. 16, 234–243. doi: 10.1111/j.1365-2702.2006.01573.x 17239058

[ref40] Reportlinker. (2020). Global Advanced Wound Care Products Industry. Available at: https://www.reportlinker.com/p05961345/global-advanced-wound-care-products-industry.html?utm_source=gnw [Accessed February 4, 2021].

[ref41] Samitsch C. (2014). Data Quality and Its Impacts on Decision-Making: How Managers Can Benefit from Good Data. London: Springer.

[ref42] Santos V. L. C. G. Oliveira A. S. Amaral A. F. S. Nishi E. T. Junqueira J. B. Kim S. H. P. (2017). Quality of life in patients with chronic wounds: magnitude of changes and predictive factors. Rev. Esc. Enferm. USP 51, 1–8. doi: 10.1590/S1980-220X2016049603250 29019529

[ref43] Sen C. K. (2021). Human wound and its burden: updated 2020 compendium of estimates. Adv. Wound Care 10, 281–292. doi: 10.1089/wound.2021.0026, PMID: 33733885 PMC8024242

[ref44] Silva L. L. D. A. T. M. Carmo C. R. M. L. (2008). Qualidade de vida dos portadores de ferida em membros inferiores: úlcera de perna. Ciencia y Enfermería. 14, 43–52. doi: 10.4067/S0717-95532008000100006

[ref1000] Sirati Nir M. Rassouli M. Ebadi A. Mosavi S. Pakseresht M. Hasan Shiri F.. (2022). Psychometric properties of the persian version of palliative care outcome scale (pos) in adult patients with cancer. Front. Psychol. 13:858684. 35602695 10.3389/fpsyg.2022.858684 PMC9122042

[ref45] Sommer R. von Stülpnagel C. C. Fife C. E. Blasingame M. Anders M. J. Thompson D.. (2020). Development and psychometric evaluation of the US English wound-QoL questionnaire to assess health-related quality of life in people with chronic wounds. Wound Repair Regen. 28, 609–616. doi: 10.1111/wrr.12837, PMID: 33372379

[ref46] Stevens J. P. (2012). Applied Multivariate Statistics for the Social Sciences. New York: Routledge.

[ref47] Terwee C. B. Bot S. D. de Boer M. R. van der Windt D. A. Knol D. L. Dekker J.. (2007). Quality criteria were proposed for measurement properties of health status questionnaires. J. Clin. Epidemiol. 60, 34–42. doi: 10.1016/j.jclinepi.2006.03.012 17161752

[ref48] The Euro Qol Group (1990). Euro Qol-a new facility for the measurement of health-related quality of life. Health Policy 16, 199–208. doi: 10.1016/0168-8510(90)90421-9 10109801

[ref49] Vowden P. Apelqvist J. Moffatt C. (2008). European Wound Management Association (EWMA). Position Document. Hard-to-Heal Wounds: A Holistic Approach. London, UK: MEP Ltd

[ref50] Werdin F. Tennenhaus M. Schaller H.-E. Rennekampff H.-O. (2009). Evidence-based management strategies for treatment of chronic wounds. Eplasty 9, 169–179. PMC2691645 19578487

[ref51] Wicke C. Bachinger A. Coerper S. Beckert S. Witte M. B. Königsrainer A. (2009). Aging influences wound healing in patients with chronic lower extremity wounds treated in a specialized wound care center. Wound Repair Regen. 17, 25–33. doi: 10.1111/j.1524-475X.2008.00438.x, PMID: 19152648

[ref52] Willis G. B. (2004). Cognitive Interviewing: A Tool for Improving Questionnaire Design. Thousand Oaks, CA: Sage Publications.

[ref53] Woo K. Santos V. G. Alam T. (2018). Optimising quality of life for people with non-healing wounds. Wounds Int. 9, 6–14.

